# Human Circulating and Tissue-Resident CD56^bright^ Natural Killer Cell Populations

**DOI:** 10.3389/fimmu.2016.00262

**Published:** 2016-06-30

**Authors:** Janine E. Melsen, Gertjan Lugthart, Arjan C. Lankester, Marco W. Schilham

**Affiliations:** ^1^Department of Pediatrics, Leiden University Medical Center, Leiden, Netherlands

**Keywords:** CD56^bright^ NK cell populations, tissue resident, lymphoid tissues, liver, uterus, NK cell development

## Abstract

Two human natural killer (NK) cell subsets are usually distinguished, displaying the CD56^dim^CD16^+^ and the CD56^bright^CD16^−/+^ phenotype. This distinction is based on NK cells present in blood, where the CD56^dim^ NK cells predominate. However, CD56^bright^ NK cells outnumber CD56^dim^ NK cells in the human body due to the fact that they are predominant in peripheral and lymphoid tissues. Interestingly, within the total CD56^bright^ NK cell compartment, a major phenotypical and functional diversity is observed, as demonstrated by the discovery of tissue-resident CD56^bright^ NK cells in the uterus, liver, and lymphoid tissues. Uterus-resident CD56^bright^ NK cells express CD49a while the liver- and lymphoid tissue-resident CD56^bright^ NK cells are characterized by co-expression of CD69 and CXCR6. Tissue-resident CD56^bright^ NK cells have a low natural cytotoxicity and produce little interferon-γ upon monokine stimulation. Their distribution and specific phenotype suggest that the tissue-resident CD56^bright^ NK cells exert tissue-specific functions. In this review, we examine the CD56^bright^ NK cell diversity by discussing the distribution, phenotype, and function of circulating and tissue-resident CD56^bright^ NK cells. In addition, we address the ongoing debate concerning the developmental relationship between circulating CD56^bright^ and CD56^dim^ NK cells and speculate on the position of tissue-resident CD56^bright^ NK cells. We conclude that distinguishing tissue-resident CD56^bright^ NK cells from circulating CD56^bright^ NK cells is a prerequisite for the better understanding of the specific role of CD56^bright^ NK cells in the complex process of human immune regulation.

## Introduction

Since the discovery of natural killer (NK) cells in 1975 (1, 2), major advances were made in deciphering the role of NK cells in health and disease. It is currently accepted that NK cells are not just “killers” that lyse infected or transformed cells but can also play an important role in modulation of immune responses due to the secretion of immunoregulatory cytokines (e.g., IFN-γ and TNF-α) and chemokines (e.g., CCL3 and CCL4). Based on this cytokine secretion profile, NK cells are classified into group 1 of the large family of innate lymphoid cells (ILCs). Developmentally, NK cells are not related to the other (non-cytotoxic) ILCs, and can be distinguished from the remaining ILCs by the expression of the transcription factor Eomesodermin (EOMES) and the cytolytic protein perforin (3, 4).

In humans, two conventional NK cell subsets have been phenotypically defined based on CD56 and CD16 (FCRγIII) surface expression: CD56^bright^CD16^−/+^ and CD56^dim^CD16^+^. While the function of CD56 [neural cell adhesion molecule (NCAM)] on NK cells is not completely understood yet, CD16 can mediate antibody-dependent cellular cytotoxicity (5). Since most research in human NK cell biology is based on peripheral blood, the herein predominant CD56^dim^ NK cell population is most extensively investigated. Based on circulating NK cells, CD56^bright^ and CD56^dim^ NK cells have usually been described as two functionally distinct subsets, cytokine producing and cytolytic, respectively. However, several observations challenge this strict difference, as that both subsets can be cytotoxic or produce cytokines, after appropriate *in vitro* stimulation. Upon target cell recognition, resting CD56^dim^ NK cells are highly cytotoxic, but can produce cytokines as well (6–8). In contrast, CD56^bright^ NK cells require monokine activation (combinations of IL2/IL12/IL15/IL18) to acquire cytolytic activity and produce cytokines (6, 9–11).

Although the CD56^dim^ NK cells predominate in blood, the CD56^bright^ NK cells are far more abundant in the human body due to their enrichment in lymphoid and non-lymphoid tissues (12–18). In addition, CD56^bright^ NK cells comprise the major NK cell population in inflamed and cancer tissues (12, 14, 19). Recently, tissue-resident CD56^bright^ NK cells were identified in liver, uterus, and lymphoid tissues, which points toward a tissue-specific function of CD56^bright^ NK cells (13, 15–17, 20–22). In order to understand the NK cell diversity, it is essential to focus on how CD56^bright^ NK cells develop, distribute, and acquire or alter their phenotype and function specifically in a particular organ. The first four developmental stages (i.e., from hematopoietic stem cell to CD56^bright^ NK cell) were already reviewed extensively elsewhere and will not be discussed here (23, 24). This review attempts to improve the understanding of human circulating and tissue-resident CD56^bright^ NK cells by reappraising their distribution and developmental, functional, and phenotypical characteristics. In addition, we will address to the developmental relationship between CD56^bright^ (stage 4) and CD56^dim^ NK cells (stage 5) and speculate on the position of tissue-resident CD56^bright^ NK cells within the NK cell developmental pathway.

## Distribution, Phenotype and Function

CD56^bright^ NK cells are widely distributed throughout the human body. When compared with blood, CD56^bright^ NK cells are enriched in most human tissues. They represent the majority of NK cells in lymph nodes, tonsil, stomach, gut, liver, uterus, adrenal gland, and visceral adipose tissue (12–18). Although CD56^bright^ NK cells seem to be outnumbered by CD56^dim^ NK cells in lung, kidney, mammillary tissue, bone marrow and spleen, this is probably a reflection of the high blood perfusion of these organs (12, 13, 18, 25). Most knowledge on the phenotype and function of CD56^bright^ NK cells is derived from blood, but it is important to realize that unique subsets of tissue-resident CD56^bright^ NK cells have been described in lymphoid tissues, liver and uterus (13, 15, 22, 26). Conceivably, more organs contain tissue-resident CD56^bright^ NK cell populations. To the best of our knowledge, no tissue-resident CD56^dim^ NK cells have been described to date. Although residency is often used as a term for organ-infiltrating NK cells, it is generally not discussed whether these NK cells are just trafficking through the organ, or truly tissue resident. In this review, we only apply the term “resident” if there is substantial evidence, which allows to distinguish the tissue-resident CD56^bright^ NK cells from circulating CD56^bright^ NK cells. The lack of CD56 expression on murine NK cells hampers the one to one comparison of CD56^bright^ NK cells to their murine counterpart. Due to limitations in obtaining human tissue samples, important findings in mice will be included in this review to cover the lack of human data.

### Hallmarks of Tissue-Resident CD56^bright^ NK Cells

In order to be retained within the tissue, tissue-resident CD56^bright^ NK cells should possess characteristics, which prevent egress from the tissue. One of the mechanisms involved in residency is attributed to CD69, which is absent from blood-derived NK cells (13). Originally, CD69 was identified as an early activation marker, but today CD69 is known to be associated with tissue residency by suppressing sphingosine-1-phospate receptor 1 (S1PR1) surface expression (27–29). Although initially identified in the context of T- and B-cell migration, S1PRs have also been proposed to mediate the egress of NK cells from tissues into blood and lymph in mice, driven by a S1P gradient (30–35). It has not been confirmed whether S1PRs are expressed as protein on the cell surface of human NK cell subsets in blood and tissues. At transcriptional level, however, both S1PR1 and S1PR5 are expressed in circulating human NK cells, with the latter being selective for CD56^dim^ NK cells (33, 36, 37). In contrast to S1PR1, S1PR5 is not inhibited by CD69 (32). Another potential mechanism for tissue homing and/or residency is the engagement of chemokine receptors. For instance, CXCR6 and CCR5 are both highly expressed on tissue-resident CD56^bright^ NK cells in lymphoid tissues and liver, but have a low expression on blood-derived CD56^bright^ NK cells, which instead express CCR7 (13, 15, 38). A third mechanism of tissue retention is driven by the expression or absence of adhesion molecules. For instance, the integrin CD49a is highly expressed on uterine CD56^bright^ NK cells, but absent from blood NK cells (20). Furthermore, tissue-resident CD56^bright^ NK cells lack CD62L (L-selectin), which is like CCR7 involved in recruitment of circulating NK cells to lymphoid tissues *via* high endothelial venules (HEVs) (15, 17, 18, 38, 39). Altogether, based on the expression of CD69, chemokine receptors, and adhesion molecules, tissue-resident CD56^bright^ NK cells can be distinguished from circulating CD56^bright^ NK cells. In addition to the phenotypical differences, tissue-resident CD56^bright^ NK cells are functionally different from their circulating counterparts as will be discussed in the next sections.

### Lymphoid Tissues

#### Lymph Node

**Figure 1:**
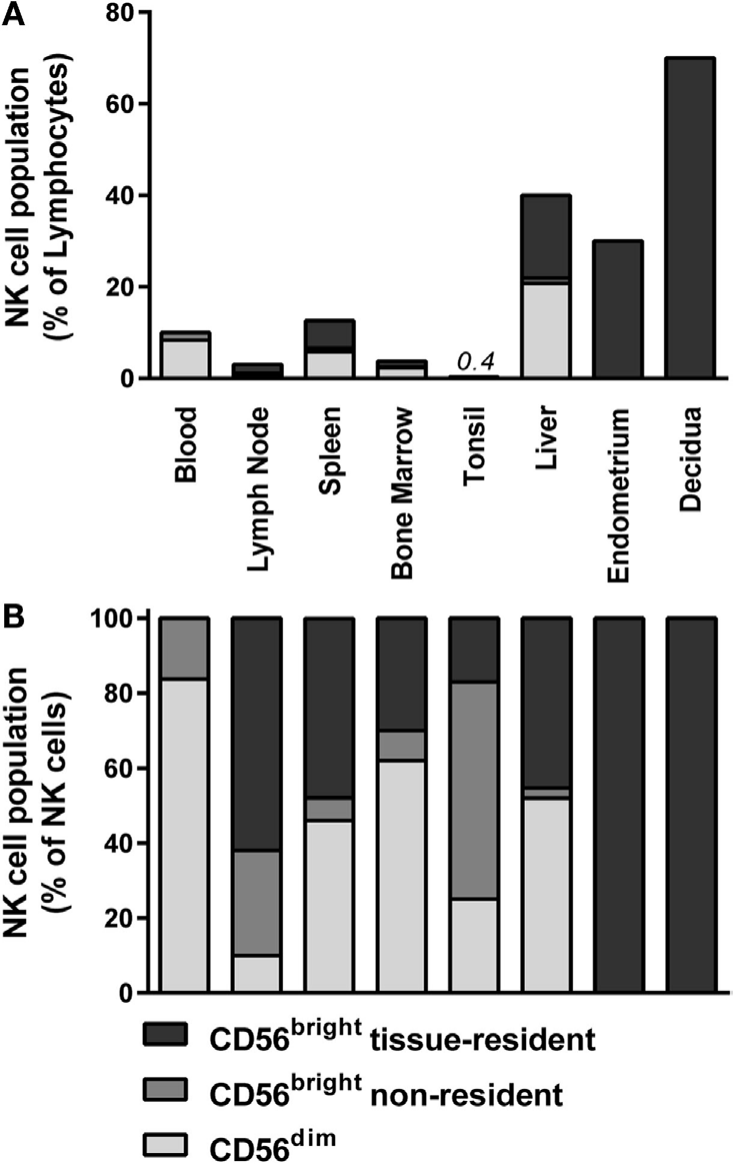
**Distribution of NK cell populations in blood and tissues**. The distribution of CD56^dim^, non-resident CD56^bright^, and tissue-resident CD56^bright^ NK cells is depicted as percentage of **(A)** total lymphocytes and **(B)** total NK cells within blood (13), lymph node (13, 18, 25), spleen (13, 18, 25), bone marrow (13, 18, 25), tonsil (18, 44), liver (15, 45, 46), endometrium (16, 20), and decidua (20, 47). Tissue-resident CD56^bright^ NK cells were defined as CD69^+^CXCR6^+^ (lymph node, spleen, and bone marrow), NKp44^+^CD103^+^ (tonsil), CD69^+^CXCR6^+^ (liver), and CD49a^+^ (endometrium and decidua). For phenotypical details, see Figure 2 and Table 1.

Lymph nodes contain 40% of the lymphocytes in the human body, of which 2–5% consist of NK cells (Figure 1A) (12, 13, 18, 40). More than 75% of the NK cells in lymph nodes have a CD56^bright^ phenotype (Figure 1B) (13, 18, 25). Accumulating evidence suggests a model in which CD56^bright^ NK cells circulate from the blood to tissues, enter the lymphatic system, and eventually migrate back to the periphery *via* the efferent lymph (41, 42). The mechanisms governing the migration to and infiltration of lymphoid tissues by CD56^bright^ NK cells are mainly deduced from chemokine receptor expression on circulating CD56^bright^ NK cells. As discussed earlier, circulating CD56^bright^ NK cells express CCR7 and CD62L (38, 39). The chemokines engaging CCR7, CCL19, and CCL21 are both highly expressed in lymph nodes (12). HEVs might not be the only route for circulating CD56^bright^ NK cells to enter the lymph node. NK cells in seroma fluid, which represents an accumulation of afferent lymph, resemble circulating CD56^bright^ NK cells regarding low expression of CCR5, killer-cell immunoglobulin-like receptor (KIR), and CD16 and high expression of CCR7 and CD62L (12, 43). This suggests that circulating CD56^bright^ NK cells enter the lymph node both *via* HEVs and afferent lymph vessels.

**Figure 2:**
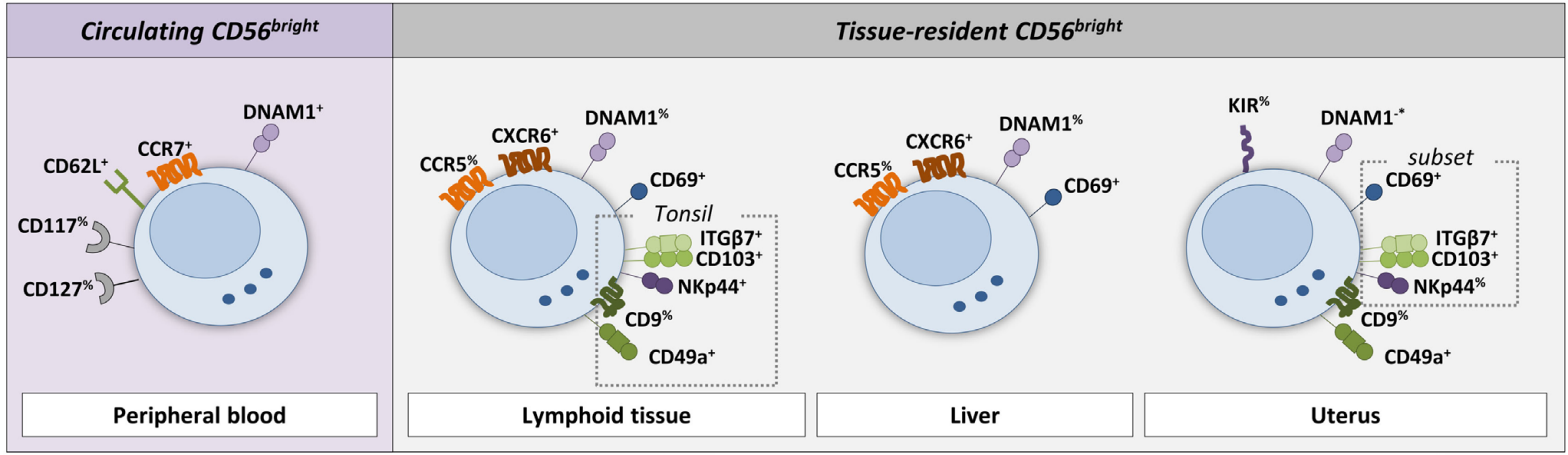
**Phenotype of circulating and tissue-resident CD56^bright^ NK cells**. The cell surface markers on NK cells that are discriminative between circulating and tissue-resident NK cells in lymphoid tissue, liver, and uterus are shown (see references in text and Table 1). Circulating CD56^bright^ NK cells typically express the lymphoid tissue homing makers CD62L (L-selectin) and CCR7. In addition, CD117 (c-kit) and CD127 (IL-7Rα) are expressed by a fraction of circulating CD56^bright^ NK cells. Lymphoid tissue-resident NK cells express CD69 and CXCR6. Tonsil-resident NK cells (defined as NKp44^+^CD103^+^) express in addition ITGβ7, CD49a, and partly CD9. The majority of CD69^+^CXCR6^+^ liver-resident NK cells express CCR5. In contrast to circulating CD56^bright^ NK cells, only a fraction of lymphoid tissue-, tonsil-, and liver-resident NK cells express DNAM1. A subset of CD49a^+^ uterus-resident NK cells (endometrium and decidua) expresses CD69, ITGβ7, CD103, and NKp44. The reported DNAM1 expression in the uterus is contradicting in the literature and therefore indicated with −*. % indicates that only a fraction of the NK cell population is positive for the marker.

Recently, we identified a major lymphoid tissue-resident NK cell subset in lymph node, spleen, and bone marrow based on co-expression of CD69 and CXCR6 (Figure 2; Table 1) (13). In the lymph node, lymphoid tissue NK (ltNK) cells account for 60% of all NK cells and cover the majority of the CD56^bright^ NK cell compartment (Figure 1B). LtNK cells display a slightly less intense CD56 and more intense NKp46 expression compared with circulating CD56^bright^ NK cells. In addition, the majority of ltNK cells is CD16^−^, CD49a^−^, and CD27^+^ (13). Interestingly, most ltNK cells do not express DNAX accessory molecule 1 (DNAM1), an activating receptor which is uniformly expressed on circulating CD56^bright^ NK cells (13). The remaining CD56^bright^CD69^−^ NK cells closely resemble circulating CD56^bright^ NK cells, suggesting that these cells are blood-derived CD56^bright^ NK cells transiently circulating through the lymph node (13).

**Table 1:** **Phenotype of circulating and tissue-resident CD56^bright^ NK cells**.

Reference	Blood (13, 15, 17, 38)	Lymph node, spleen, marrow (13, 18)	Tonsil (18, 44)	Liver (15, 45, 46)	Uterus (16, 17, 20, 48, 49)	
Definition		CD69^+^ CXCR6^+^	NKp44^+^ CD103^+^	CD69^+^ CXCR6^+^	CD49a^+^ CD103^−^	CD49a^+^ CD103^+^

CD56	++	+	+/++	+	+++	+++
CD69	–	+	+	+	–	+
**Cytokine receptors**						
CD117 (c-kit)	%	–	N.A.	N.A.	–	–
CD127 (IL7-Rα)	%	–	–	N.A.	–	–
**Chemokine receptors**						
CCR7	+	–	–	–	–	–
CCR5	–	%	N.A.	%	–	–
CXCR6	–	+	+	+	N.A.	N.A.
**NK cell receptors**						
DNAM1	+	%	%	%	–^a^	–^a^
KIR	–	–	%	–	%	%
NKp44	–	–	+	–	–	%
NKp46	+	++	++	++	+	+
**Adhesion molecules**						
CD9	–	N.A.	%	N.A.	%	+
CD49a (ITGα1)	–	–	+	–	+	+
CD62L (L-selectin)	+	–	–	–	–	–
CD103 (ITGαE)	–	–	+	N.A.	–	+
ITGβ7	N.A.	N.A.	+	N.A.	–	+

*The cell surface markers which are discriminative for circulating CD56^bright^ NK cells, lymph node-, spleen-, bone marrow-, tonsil-, liver-, and uterus-resident NK cells are summarized. Uterus-resident NK cells can be subdivided based on CD103 expression*.

*++ and +++ indicate relatively higher levels of expression*.

*% indicates that only a fraction of the NK cell population is positive for the marker*.

*^a^Contradicting literature exist on the DNAM expression on uterine NK cells*.

*N.A., not assessed*.

Lymphoid tissue NK cells were tested in the functional assays classically used for NK cells. The ltNK cells were less potent IFN-γ producers compared with circulating CD56^bright^ NK cells nor did they lyse K562 target cells as efficient as CD56^dim^ NK cells (13). However, the expression of EOMES and perforin distinguishes ltNK cells from the helper-ILC1 group (13). These phenotypical and functional characteristics, combined with their specific location in lymphoid tissues where immune responses are initiated and shaped, point to a distinct yet undefined role of ltNK cells (50).

Notably, without the use of tissue-resident markers, CD69 and CXCR6, ltNK cells could previously not be distinguished from circulating CD56^bright^ NK cells. Therefore, a re-examination of the function of CD56^bright^ NK cells in lymphoid tissue is necessary, in particular in the lymph node where a large population of ltNK cells co-exists next to the non-resident or circulating CD56^bright^ NK cells.

#### Spleen and Marrow

The spleen and bone marrow contain 14 and 10% of the total lymphocyte pool. NK cells constitute 5–20% and 4% of lymphocytes in spleen and marrow, respectively (Figure 1A) (13, 18, 40, 43). The CD56^bright^ and CD56^dim^ NK cells are equally distributed in the spleen, but 90% of CD56^bright^ NK cells consist of ltNK cells (Figure 1B) (13, 25, 40). Similar to the spleen, the bone marrow is enriched for CD56^bright^ NK cells, of which the majority consists of ltNK cells (13, 25). The phenotype of ltNK cells in spleen and marrow resembles the ltNK cell population in lymph node. As mentioned before, the non-resident CD56^bright^CD69^−^ and CD56^dim^ NK cells in marrow and spleen closely resemble the circulating CD56^bright^ and CD56^dim^ NK cells and are probably circulating NK cells contained in the tissue at time of isolation. Previously, the spleen has been reported to be enriched in CD27^+^ and NKp46^bright^ NK cells, which could be a reflection of ltNK cells. These findings further illustrate the importance of using tissue-resident markers to distinguish circulating from tissue-resident CD56^bright^ NK cells (51, 52). The manner in which NK cells enter the spleen differs from lymph node entrance, because the spleen does not contain afferent lymphatic vessels or HEVs (53). In mice, NK cells enter the spleen *via* arterioles in the marginal zone, rather than *via* arterioles directly connected to the red pulp, where most NK cells reside (54). Unfortunately, there is a lack of human studies focusing on how NK cells migrate to the spleen and bone marrow.

#### Tonsil

In tonsil, although the CD56^bright^ subset is predominant, only 0.4% of the total lymphocytes consist of NK cells (Figure 1A) (18). Seventeen percent of the total NK cell population in the tonsil co-expresses CD69 and CXCR6 (Figure 1B) (44). In contrast to the ltNK cells in lymph node, marrow, and spleen, these tonsil-resident NK cells also express NKp44, CD103, CD49a, Integrinβ7, and partly CD9 (Figure 2; Table 1) (44). Of note, tonsil-resident NK cells should be distinguished from NKp44^+^ ILC3s, which are located in the mucosa surrounding the lymphoid follicles and secrete preferentially IL-22 (55, 56). Similar to circulating CD56^bright^ NK cells, the total pool of CD56^bright^ NK cells was shown to produce high levels of IFN-γ and to become cytolytic upon IL-2 and/or IL-12 stimulation (18). Tonsils do not have afferent lymph vessels but HEVs are present, which might support the trafficking of NK cells. Similarly, CCL19 and CCL21 are secreted to attract circulating CD56^bright^ NK cells, which might explain the high content of CD56^bright^ NK cells which lack a tissue-resident phenotype (57).

### Liver

Hepatic NK cells comprise 40% of all hepatic lymphocytes (Figure 1A) (45). Recently, a major liver-resident EOMES^+^CD56^bright^ NK cell population has been described, which comprises 45% of the hepatic NK cells and closely resembles ltNK cells phenotypically and functionally (Figure 1B) (15, 45, 46). Liver-resident CD56^bright^ NK cells are characterized by a simultaneous expression of CD69 and CXCR6 (46). They have a high expression of CCR5 and NKp46, and low expression of DNAM1, as indirectly concluded from phenotypical analysis on total hepatic CD56^bright^ NK cells (Figure 2; Table 1) (15, 45).

An independent report demonstrated the presence of a distinct minor liver-resident cell population characterized by CD49a expression (21). Those CD56^bright^CD49a^+^ cells make up 2% of the total NK cell compartment in the liver but are not present in every individual (41% of donors) (13, 15, 21). The expression of CXCR6 has not been described. However, these cells do not express EOMES, suggesting that they do not belong to the NK cell lineage (21). Due to the low prevalence of this CD49a^+^EOMES^−^ cell population, we can indirectly conclude that the major CD69^+^CXCR6^+^ liver-resident NK cell population is negative for CD49a. The IFN-γ production of liver-resident CD56^bright^ NK cells after 4-h stimulation with IL12 and IL18 was lower compared with the non-resident hepatic NK cells (46). Similar to ltNK cells, liver-resident CD56^bright^ NK cells express perforin and granzyme B at a low level, further supporting a non-cytotoxic function (46).

Several studies in mice demonstrated the existence of hapten and virus-specific hepatic NK cell memory, mediated by cells expressing CD49a and CXCR6 (21, 58, 59). In contrast, splenic CXCR6^+^ NK cells, which potentially resemble the human ltNK cells, were not able to mediate a memory response (58). Thus, although CXCR6 expression is not restricted to the liver, only hepatic NK cells were found to mediate a memory response in mice. Nevertheless, it would be interesting to further study the memory capacities of the highly prevalent CXCR6^+^ liver- and lymphoid tissue-resident CD56^bright^ NK cells in humans.

### Uterus

The uterine mucosa is populated by EOMES^+^CD56^bright^ NK cells (16, 17, 23). In contrast to blood, there are hardly any CD56^dim^ NK cells detectable in endometrium (no pregnancy) and decidua (pregnancy) (16, 17, 20, 60). Independent of the stage of the menstrual cycle, NK cells make up 30% of the endometrial lymphocytes (Figure 1A), although the absolute number of lymphocytes and NK cells increases robustly in the secretory stage (16). During early pregnancy, however, more than 70% of the lymphocytes in the uterine decidua is represented by CD56^bright^ NK cells (Figure 1A) (47). Phenotypically, endometrial and decidual CD56^bright^ NK cells closely resemble each other, and will be further referred to as uterine CD56^bright^ NK cells. The CD56 expression of the uterine NK cells is even more intense than their circulating CD56^bright^ counterparts (Table 1) (17, 61). All uterine NK cells display CD49a but not CCR5, discriminating them from the circulating, lymphoid tissue, and liver-resident NK cell populations (Figure 2) (16, 20). DNAM1 has been reported to be absent on uterine NK cells, although a contradicting report on this observation exists (20, 48, 62). Recently, it was shown that a fraction of uterine NK cells expresses the heterodimer CD103/ITGβ7, NKp44, as well as CD69 (20, 62). Conversely, an earlier study reported that all decidual NK cells express CD69 (17). Despite this discrepancy concerning the CD69 expression, both the CD56^bright^CD103^−^ and CD56^bright^CD103^+^ NK cells are likely to represent a tissue-resident CD56^bright^ NK cell population, as demonstrated by the expression of KIRs, CD9, and poor IFN-γ production and cytotoxicity (16, 17, 20, 49, 63, 64). Moreover, transcriptome analysis of decidual NK cells and circulating NK cells highlighted the uniqueness of the uterine NK cells (17, 48). To the best of our knowledge, the presence of chemokine receptors, such as CXCR6 and CCR5, has not been reported. Compared with circulating CD56^bright^ NK cells, decidual CD56^bright^ NK cells highly express the activating receptors NKG2C and NKG2E at RNA level; however, <30% is NKG2C^+^ on protein level (17, 62, 65).

Initially, a suppressive function of decidual NK cells was thought to be essential to provide maternal–fetal tolerance (17, 64). However, accumulating evidence points toward a more active role of decidual CD56^bright^ NK cells in regulating placentation, as reviewed elsewhere (66). Decidual NK cells are considered to stimulate trophoblast invasion and spinal artery remodeling *via* the production of various chemokines and angiogenic factors (including angiopoietins and GM-CSF) (67–69). Mice lacking decidual NK cells exhibit abnormalities in pregnancy, including abnormal vascular remodeling of decidual arteries (70). Although the process of placentation in humans is different, specific combinations of fetal HLA-C alleles, presented by trophoblasts, and maternal KIR expression were shown to be associated with successful placentation (69, 71). The similarities between endometrial and decidual CD56^bright^ NK cells suggest that decidual CD56^bright^ NK cells are a direct reflection of endometrial CD56^bright^ NK cells in a pregnant tissue microenvironment. Taken together, the phenotypical and functional profile of the uterine CD56^bright^ NK cell compartment supports their unique functional role during pregnancy.

## Developmental Relationship Between Circulating CD56^dim^ and CD56^bright^ NK Cells

Thus far, we discussed the tissue-resident and circulating/non-resident CD56^bright^ NK cells within the tissues. Still, the origin of the different CD56^bright^ NK cell populations and their relation to the CD56^dim^ NK cell subset remains unclear. The circulating CD56^bright^ NK cells have been extensively investigated and are generally considered to be the precursors of the CD56^dim^ NK cells. In the last section of this review, we will summarize the current evidence in favor and against the linear relationship between the circulating CD56^bright^ and CD56^dim^ NK cells, and speculate on the position of tissue-resident NK cells in this developmental pathway.

Several studies provided clues about the developmental relationship between CD56^bright^ NK cells and CD56^dim^ NK cells. First, it was shown that CD56^bright^, but not CD56^dim^ NK cells, constitutively express the high-affinity IL-2Rα (CD25) and display a high proliferative response in the presence of picomolar concentrations of IL-2 (72, 73). Because CD56^bright^ NK cells have significantly longer telomeres compared with CD56^dim^ NK cells, they have been assumed to have a shorter proliferative history (74). A commonly used marker for immaturity, the tyrosine kinase c-kit (receptor for stem cell factor, CD117) is expressed on a fraction of CD56^bright^ NK cells, but is absent on CD56^dim^ NK cells (75, 76). In addition, the recovery of CD56^bright^ NK cells in the first weeks after hematopoietic stem cell transplantation (HSCT) precedes the reconstitution of CD56^dim^ NK, a sequential occurrence potentially pointing toward a developmental relationship (74, 77). Together, these findings resulted in the hypothesis that CD56^dim^ NK cells are derived from CD56^bright^ NK cells.

### Differentiation from CD56^bright^ to CD56^dim^ NK Cells *In Vitro*

In efforts to provide evidence for this hypothesis, numerous studies aimed to recapitulate the differentiation from CD56^bright^ to CD56^dim^ NK cells *in vitro*. CD56^bright^ NK cells were shown to acquire a CD56^dim^-like phenotype upon *in vitro* activation with IL-2, IL-15, and/or co-culture with T cells. This resulted in the upregulation of CD16 and KIRs and the downregulation of IL-7Rα (CD127), CD117, CXCR3, and CCR7 (10, 74). However, the intensity of CD56 expression was not reduced on monokine-activated CD56^bright^ NK cells. The presence of fibroblast growth factor receptor 1 (FGFR1) was demonstrated to be critical for the *in vitro* differentiation of CD56^bright^ NK cells to cytotoxic CD56^dim^ NK cells in a contact-dependent manner (78). FGFR1 is a ligand for CD56 and is constitutively expressed on fibroblasts (79, 80). The high density of CD56 on CD56^bright^ NK cells may thus be of importance in the interaction with fibroblasts and differentiation toward CD56^dim^ NK cells.

### Differentiation from CD56^bright^ to CD56^dim^ NK Cells *In Vivo*

The *in vivo* evaluation of the relationship between CD56^bright^ and CD56^dim^ NK cells is hampered by the lack of CD56 expression on murine NK cells. The vast majority of human CD56^bright^ NK cells displayed a reduction of CD56 expression intensity after infusion into immune-deficient mice (78). Whether these *in vivo* differentiated CD56^dim^ NK cells were phenotypically and functionally similar to human blood-derived CD56^dim^ NK cells were not addressed in this study, leaving the possibility that the bright CD56 expression is not sustained in mice lacking human fibroblasts expressing FGFR1. An alternative for murine experiments can be provided by the study of rhesus macaques. Gene tracking data in rhesus macaques transplanted with lentiviral barcoded hematopoietic stem cells demonstrated that the lineage origin of the macaque NK cell homologs of CD56^bright^ (CD56^+^CD16^−^) and CD56^dim^ (CD56^−^CD16^+^) NK cells is different (81). While the CD56^bright^ homolog was derived from the same progenitors as T-cell, B-cell, and myeloid cells, the CD56^dim^ homolog displayed a unique clonal pattern, suggesting that these cells do not develop from the CD56^bright^ population but may belong to an independent lineage (81).

In addition to mice and macaques studies, human NK cell deficiencies can provide clues about the developmental relationship between CD56^bright^ and CD56^dim^ NK cells. Mutations in the transcription factor gene *GATA2* result in the absence of CD56^bright^ NK cells while CD56^dim^ NK cells are still present (82). This observation argues against the theory that CD56^dim^ NK cells are derived from CD56^bright^ NK cells. On the other hand, humans with a partial minichromosome maintenance complex 4 (MCM4) deficiency, a molecule involved in proliferation, have reduced numbers of circulating CD56^dim^ NK cells but normal numbers of CD56^bright^ NK cells (83). This could indicate that maintenance of the CD56^dim^ NK cell subset requires proliferation, which might be dependent or independent of the CD56^bright^ NK cells. To the best of our knowledge, there are no mutations in transcription factors described, which cause a lack of CD56^dim^ NK cells while the CD56^bright^ NK cells are spared. Recently, Zeb2 was identified as the essential regulator of terminal NK cell maturation in mice and shown to be higher expressed in circulating CD56^dim^ compared with CD56^bright^ NK cells (84). Together, these studies emphasize the need for additional experimental evidence on the transcriptional regulation of human NK cell development.

### Intermediate Stages Connecting CD56^bright^ to CD56^dim^ NK Cells

If CD56^bright^ and CD56^dim^ NK cells are successive stages in the NK cell developmental pathway, developmental intermediates should exist. Independent studies reported the existence of phenotypic and functional intermediate stages in the progression from CD56^bright^ to CD56^dim^ NK cells in peripheral blood of healthy donors and patients after HSCT. These studies mainly focused on CD16, CD27, or CD117, for which CD56^bright^ NK cells have a bimodal expression profile (10, 51, 85–88). Both CD16^+^ and CD27^−^ CD56^bright^ NK cells were independently suggested to represent intermediate populations based on phenotype and functional characteristics (10, 51, 88). A relative increase of CD16^+^, CD27^−^, and CD117^−^ CD56^bright^ NK cells was observed early after HSCT (85, 86). Notably, the expression of CD16, CD117, and CD27 on CD56^bright^ NK cells can also be modulated by cytokine-activation (10, 51, 74). Because the post-HSCT setting presents a cytokine-rich environment, the “intermediate” CD16^+^, CD27^−^, and CD117^−^ CD56^bright^ NK cells may represent cytokine-activated CD56^bright^ NK cells instead of developmental intermediates between CD56^bright^ and CD56^dim^ NK cells (85, 86).

In general, the potential differentiation of circulating CD56^bright^ NK cells to CD56^dim^ NK cells is characterized by loss of CD27, CD117, NKG2A, and CD62L expression and gain of CD16, KIRs, and CD57 expression. Both within the CD56^bright^ compartment (CD117↓, CD27↓, and CD16↑) as well as within the CD56^dim^ compartment (NKG2A↓, CD62L↓, KIRs↑, and CD57↑), the sequential loss and acquisition of these surface markers do not occur in a fixed order (10, 51, 87, 89, 90). Only the extremes of these markers, for instance CD117 and CD57, are mutually exclusively expressed. Together, this illustrates that uniform intermediate stages of differentiation between CD56^bright^ and CD56^dim^ NK cells cannot easily be identified.

## Developmental Position of Tissue-Resident CD56^bright^ NK Cells

Studies on the relationship between the NK cell populations have been based on blood-derived CD56^bright^ and CD56^dim^ NK cells. The starting point of most of these studies was a linear developmental relationship between CD56^bright^ and CD56^dim^ NK cells. However, the discovery of distinct tissue-resident CD56^bright^ NK cell populations increases the number of possible relationships between the NK cell populations. Tissue-resident NK cells could be a precursor to circulating NK cells, but the absence of the immature markers CD117 and CD127 argues against this. It also seems unlikely that tissue-resident NK cells represent a transitory population between the circulating CD56^bright^ and CD56^dim^ NK cells. Detailed transcriptome analysis comparing uterine NK cells with both circulating CD56^bright^ and CD56^dim^ NK cells highlighted major differences in gene expression profile between the three NK cell populations (17). Moreover, data from transcription factor-deficient mice suggested that circulating and tissue-resident NK cells are derived from different cell lineages (91). In our opinion, the distinct phenotype and functional signature of the tissue-resident NK cell populations, together with their absence from blood, argues in favor of the hypothesis that tissue-resident CD56^bright^ NK cells develop locally, independently of the circulating NK cells. It seems likely that the organ microenvironment is essential to induce the phenotype and retain tissue localization of tissue-resident cells. Nevertheless, additional studies are needed to shed new light on the developmental relationship between CD56^bright^, CD56^dim^ and tissue-resident CD56^bright^ NK cell populations.

## Concluding Remarks

The recent identification of tissue-resident CD56^bright^ NK cells in the lymphoid tissues, liver, and uterus led us to reappraise the characteristics of CD56^bright^ NK cell populations in the circulation and tissues. The function of tissue-resident CD56^bright^ NK cells in liver and lymphoid tissues has not been elucidated, although it is very likely that these cells, such as uterine NK cells, exert tissue-specific functions.

The existence of tissue-resident NK cells raises the question whether, and if so how, all the NK cell populations are developmentally related to each other. Based on the available evidence, we conclude that it is still possible that CD56^dim^ NK cells develop independently from the CD56^bright^ NK cells. Tissue-resident NK cells may develop from circulating CD56^bright^ NK cells, or follow their own developmental pathway. Current *in vitro* models do not sufficiently mimic the *in vivo* situation, especially considering the potentially important role of the tissue microenvironment in shaping the features of tissue-resident CD56^bright^ NK cells. As mouse models do not suffice in the evaluation of human NK cell subsets, other animal models might be exploited. Studying patients with aberrations in NK cell development due to genetic mutations could provide novel insights in the origin and development of tissue-resident NK cells. Furthermore, transcriptome analysis of non-resident and resident CD56^bright^ NK cell populations will provide tools to further decipher the role of CD56^bright^ NK cell populations in human immune responses. In conclusion, distinguishing tissue-resident CD56^bright^ NK cells from circulating CD56^bright^ NK cells is a prerequisite for the better understanding of the specific role of CD56^bright^ NK cells in the complex process of human immune regulation.

## Author Contributions

JM and GL wrote the manuscript. AL and MS critically revised the manuscript and approved it for publication.

## Conflict of Interest Statement

The authors declare that the research was conducted in the absence of any commercial or financial relationships that could be construed as a potential conflict of interest.

## Abbreviations

DNAM1, DNAX accessory molecule 1; EOMES, eomesodermin; FGFR1, fibroblast growth factor receptor 1; HEVs, high endothelial venules; HSCT, hematopoietic stem cell transplantation; ILC, innate lymphoid cell; KIR, killer-cell immunoglobulin-like receptor; ltNK cells, lymphoid tissue natural killer cells; MCM4, minichromosome maintenance complex 4; S1PR1, sphingosine-1-phospate receptor 1.
